# Heat-Stress Responses Differ among Species from Different ‘*Bangia*’ Clades of Bangiales (Rhodophyta)

**DOI:** 10.3390/plants10081733

**Published:** 2021-08-22

**Authors:** Ho Viet Khoa, Puja Kumari, Hiroko Uchida, Akio Murakami, Satoshi Shimada, Koji Mikami

**Affiliations:** 1 Graduate School of Fisheries Sciences, Hokkaido University, 3-1-1 Minato-cho, Hakodate 041-8611, Japan; hvkhoa59@gmail.com; 2 Faculty of Fisheries Sciences, Hokkaido University, 3-1-1 Minato-cho, Hakodate 041-8611, Japan; pujamashal@gmail.com; 3 School of Biological Sciences, University of Aberdeen, Cruickshank Building, St. Machar Drive, Aberdeen AB24 3UU, UK; 4 Kobe University Research Center for Inland Seas, 2746 Iwaya, Awaji 656-2401, Japan; ucchy@maia.eonet.ne.jp (H.U.); akiomura@kobe-u.ac.jp (A.M.); 5 Graduate School of Science, Department of Biology, Kobe University, 1-1 Rokkodai-cho, Nada-ku, Kobe 657-8501, Japan; 6 Department of Biology, Ochanomizu University, 2-1-1 Otsuka, Bunkyo-ku, Tokyo 112-8610, Japan; satoshimiru@gmail.com; 7 Department of Integrative Studies of Plant and Animal Production, School of Food Industrial Sciences, Miyagi University, 2-2-1 Hatatate, Taihaku-ku, Sendai 982-0215, Japan

**Keywords:** *Bangia atropurpurea*, ‘*Bangia*’ sp., heat stress, asexual reproduction, stress memory, thermotolerance

## Abstract

The red alga ‘*Bangia*’ sp. ESS1, a ‘*Bangia*’ 2 clade member, responds to heat stress via accelerated asexual reproduction and acquires thermotolerance based on heat-stress memory. However, whether these strategies are specific to ‘*Bangia*’ 2, especially ‘*Bangia*’ sp. ESS1, or whether they are employed by all ‘*Bangia*’ species is currently unknown. Here, we examined the heat-stress responses of ‘*Bangia*’ sp. ESS2, a newly identified ‘*Bangia*’ clade 3 member, and *Bangia atropurpurea.* Intrinsic thermotolerance differed among species: Whereas ‘*Bangia*’ sp. ESS1 survived at 30 °C for 7 days, ‘*Bangia*’ sp. ESS2 and *B. atropurpurea* did not, with *B. atropurpurea* showing the highest heat sensitivity. Under sublethal heat stress, the release of asexual spores was highly repressed in ‘*Bangia*’ sp. ESS2 and completely repressed in *B. atropurpurea*, whereas it was enhanced in ‘*Bangia*’ sp. ESS1. ‘*Bangia*’ sp. ESS2 failed to acquire heat-stress tolerance under sublethal heat-stress conditions, whereas the acquisition of heat tolerance by priming with sublethal high temperatures was observed in both *B. atropurpurea* and ‘*Bangia*’ sp. ESS1. Finally, unlike ‘*Bangia*’ sp. ESS1, neither ‘*Bangia*’ sp. ESS2 nor *B. atropurpurea* acquired heat-stress memory. These findings provide insights into the diverse heat-stress response strategies among species from different clades of ‘*Bangia*’.

## 1. Introduction

Bangiales is a monophyletic order of red algae [1] comprising over 150 species [2]. Although filamentous *Bangia* Lyngb. and foliose *Porphyra* C. Agardh were previously recognized as genera within Bangiales [3], recent phylogenetic analyses demonstrated the presence of unexpected diversity in Bangiales, with cryptic species showing highly similar morphologies [4,5,6,7,8,9]. This limits the taxonomic analysis of Bangiales based on morphological characteristics. Therefore, to understand the diversity within Bangiales, it is important to compare the nucleotide sequences of widely conserved genes, such as the plastid gene *rbcL*, encoding the large subunit of ribulose 1,5-bisphosphate carboxylase/oxygenase (Rubisco), and the nucleus-encoded small subunit ribosomal ribonucleic acid (SSU rRNA).

Based on phylogenetic analyses of *rbcL* and SSU rRNA [10,11,], the taxonomy of Bangiales was revised by splitting it into 13 genera (including 4 filamentous and 9 foliose genera) and 3 filamentous clades. Accordingly, although for the past few decades, all members of the genus *Bangia* were considered to include only three species, i.e., *B. atropurpurea*, *B. fuscopurpurea*, and *B. gloiopeltidicola*, the previously recognized genus *Bangia* was recently divided into one genus (*Bangia*) and three clades (‘*Bangia*’ 1, ‘*Bangia*’ 2, and ‘*Bangia*’ 3) [10]. In this classification system, *B. atropurpurea* and *B. gloiopeltidicola* fall into *Bangia* and ‘*Bangia*’ 3, respectively, while *B. fuscopurpurea*, which has been used in numerous studies and is thought to represent a single species, is classified into ‘*Bangia*’ 2 and ‘*Bangia*’ 3 [10]. Thus, species previously classified as *B. fuscopurpurea* are a mixture of phylogenetically close but distinct species.

In Japan, species belonging to ‘*Bangia*’ are widely distributed along coastal regions. However, the local and seasonal distributions of these species have not been extensively surveyed. It is therefore unclear whether species belonging to the three ‘*Bangia*’ clades are present in Japan, although the ‘*Bangia*’ 2 species, ‘*Bangia*’ sp. ESS1, has been identified in Esashi, Hokkaido [12]. The use of a combination of molecular phylogenetic and physiological approaches to study species from various locations could potentially resolve this question.

The physiological properties of ‘*Bangia*’ species were recently investigated, specifically the response to heat stress. For instance, heat-inducible asexual reproduction to produce gametophytic clones via the release of asexual spores from gametophytes was observed in ‘*Bangia*’ sp. ESS1 and other species collected from various locations [13,14,15]. In addition, ‘*Bangia*’ sp. ESS1 can remember heat stress, allowing it to survive subsequent exposure to lethal temperatures after priming via exposure to sublethal temperatures [16]. This indicates that these algae maintain thermotolerance as a heat stress-responsive physiological state during subsequent non-stress control conditions, which increases the threshold level of heat-stress sensing. These findings suggest that ‘*Bangia*’ sp. ESS1 responds to heat stress by producing new generations via accelerated asexual reproduction and the acquisition of thermotolerance based on stress memory. However, it is currently unknown whether these strategies are specific to ‘*Bangia*’ 2, especially ‘*Bangia*’ sp. ESS1, or whether they are common to all ‘*Bangia*’ species.

Here, to address this question, we identified the marine species living at the rocky coast ‘*Bangia*’ sp. ESS2, a Bangiales belonging to ‘*Bangia*’ 3, and characterized its heat-stress responses in terms of growth-limiting temperature, accelerated asexual reproduction, and the acquisition of heat-stress tolerance compared to ‘*Bangia*’ sp. ESS1 [12,16] and the freshwater species living close to the mountain stream *B. atropurpurea* [17]. Our results uncover the diverse heat-stress response strategies among ‘*Bangia*’ species from different ‘*Bangia*’ clades.

## 2. Results and Discussion

### 2.1. Identification of a Species Belonging to ‘Bangia’ 3

**Figure 1:**
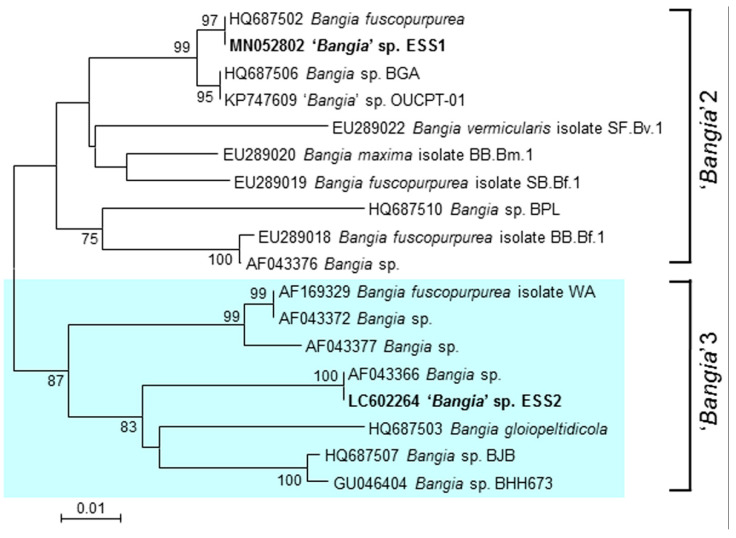
Phylogenetic identification of ‘*Bangia*’ sp. ESS2. The phylogenetic tree was constructed by the neighbor-joining method using sequences of *rbcL* genes from different species from the ‘*Bangia*’ 2 and ‘*Bangia*’ 3 groups identified by Sutherland et al. [10]. The DDBJ/EMBL/GenBank accession numbers of the *rbcL* gene sequences are shown in front of the species names. ‘*Bangia*’ sp. ESS1 and ‘*Bangia*’ sp. ESS2 are highlighted. Bootstrap values over 50% from 1000 replicates are indicated at the nodes. Bar, 0.01 substitutions per site.

We amplified and sequenced a DNA fragment corresponding to the *rbcL* gene from gametophytes of a species collected on Kamomejima Island, Esashi, in April 2018 (GenBank accession number LC602264). We then performed phylogenetic analysis using the *rbcL* sequences from this species and species belonging to ‘*Bangia*’ 2, including ‘*Bangia*’ sp. ESS1 [12] and ‘*Bangia*’ sp. OUCPT-01 [18], as well as the ‘*Bangia*’ 3 clade of Bangiales [10]. As shown in Figure 1, the species was classified as a member of ‘*Bangia*’ 3, with the closest relationship to *Bangia* sp. collected on Disko Island in Greenland, Rankin Inlet in Canada, and Chaichei Island in the United States, whose *rbcL* sequences were identical and deposited in GenBank under accession number AF043366 [19]. We designated the new species ‘*Bangia*’ sp. ESS2 (ESS represents Esashi).

*Bangia gloiopeltidicola*, a well-known epiphytic seaweed of the red alga *Gloiopeltis furcata* (family Endocladiaceae), is a typical ‘*Bangia*’ 3 species. However, ‘*Bangia*’ sp. ESS2 adheres to rocks in the intertidal zone, pointing to the diversity of lifestyle strategies among ‘*Bangia*’ 3 members.

### 2.2. Morphological and Developmental Properties of ‘Bangia’ sp. ESS2

**Figure 2:**
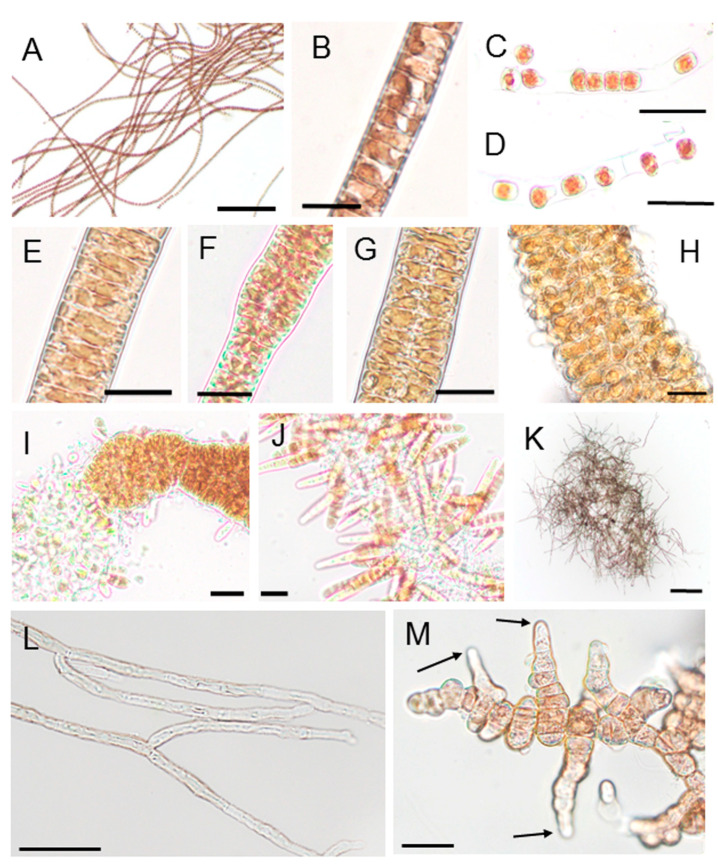
Morphological characteristics of thalli, conchocelis filaments, and conchosporangia of ‘*Bangia*’ sp. ESS2: (**A**,**B**) Filamentous structure of gametophytic thalli; (**C**,**D**) Release of asexual spores from uniseriate thalli; (**E**–**H**) Asexual sporangium development, including doubling of the width of the vegetative cells, vertical cell division to form biseriate thalli, and random cell division to form multiseriate thalli; (**I**) Release of asexual spores from the tip of a multiseriate thallus; (**J**) Development of gametophytic thalli on a parental thallus without spore release; (**K**) Aggregate of conchocelis filaments, which appear black; (**L**) Magnified view of conchocelis filaments with branches; (**M**) Conchosporangia produced on conchocelis filaments. Many branches were produced in conchosporangia, unlike conchocelis filaments, which formed branches only infrequently; the pointed tip cells are indicated by arrows. Scale bars: 25 μm for A–J and M, 200 μm for K, 50 μm for L.

Thalli of ‘*Bangia*’ sp. ESS2 (Figure 2A) were usually uniseriate or biseriate filaments containing cylindrical vegetative cells (Figure 2B,G, respectively). The uniseriate filaments were 10.58 ± 0.85 μm in diameter (*N* = 20) and 8.65 ± 1.52 μm long (*N* = 26), and the biseriate filaments were 20.87 ± 2.12 μm in diameter (*N* = 20) and 6.445 ± 1.52 μm long (*N* = 26). Asexual spores, which are called monospores or archeospores, were released from both uniseriate and multiseriate thalli. When asexual spores were released from uniseriate thalli, the vegetative cells developed into asexual spores in the thalli and were released by rupture of the cell wall (Figure 2C,D). Release of asexual spores from multiseriate thalli required that uniseriate thalli develop into multiseriate thalli also known as asexual sporangia (Figure 2E–J). In this process, the vegetative cells grew to approximately twice as wide (Figure 2E) as uniseriate gametophytes (Figure 2B), and vertical cell division occurred, leading to the formation of a biseriate filament (Figure 2F,G). Subsequently, the number of vegetative cells in the filaments increased due to cell division, while the size of the cells decreased, resulting in the generation of asexual sporangia (Figure 2H). The asexual spores were released from the tip of the sporangium or developed into gametophytes without being released, to form gametophytic clones (Figure 2I,J).

It was difficult to induce male and female gamete development under laboratory culture conditions. However, some naturally harvested thalli had already formed carposporangia via the fertilization of male and female gametes. Thus, these carposporangia produced conchocelis filaments in the laboratory (Figure 2K), which appeared black. Thus, it is clear that ‘*Bangia*’ sp. ESS2 undergoes both sexual and asexual propagation during its life cycle. As shown in Figure 2L, all conchocelis filaments were uniseriate, with cylindrical cells (4.62 ± 0.72 μm in diameter (*N* = 20)/12.76 ± 1.65 μm long (*N* = 27)), from which branches were often produced. Conchosporangia developed on the conchocelis filaments as thick filaments composed of cells 16.97 ± 2.97 μm in diameter (*N* = 27) and 15.37 ± 2.69 μm long (*N* = 26), which underwent branching (Figure 2M). These findings indicate that the morphologies of ‘*Bangia*’ sp. ESS2 are similar to those of other previously reported ‘*Bangia*’ species [13,14].

Notably, the tip of each conchosporangium was pointed (Figure 2M), which is similar to the pointed conchosporangium tips of a *Porphyra* species collected in New Zealand [20]; however, other known *Neopyropia* species such as *N. pseudolinearis* and *N. yezoensis* have rounded tips [21,22]. Thus, as mentioned in Knight and Nelson [20], it appears that the morphology and shape of conchosporangia are effective taxonomic characteristics for discovering new species of Bangiales, although molecular validation via phylogenetic analysis would provide indispensable confirmatory evidence. Indeed, the conchosporangium tips of ‘*Bangia*’ sp. collected in Fukaura, Aomori in Japan were pointed (see Figure 1C in [13]), suggesting that this species might be a ‘*Bangia*’ 3 species, like ‘*Bangia*’ sp. ESS2.

### 2.3. Growth-Limiting Temperatures of Gametophytic Thalli in ‘Bangia’ sp. ESS2 and Bangia Atropurpurea

**Figure 3:**
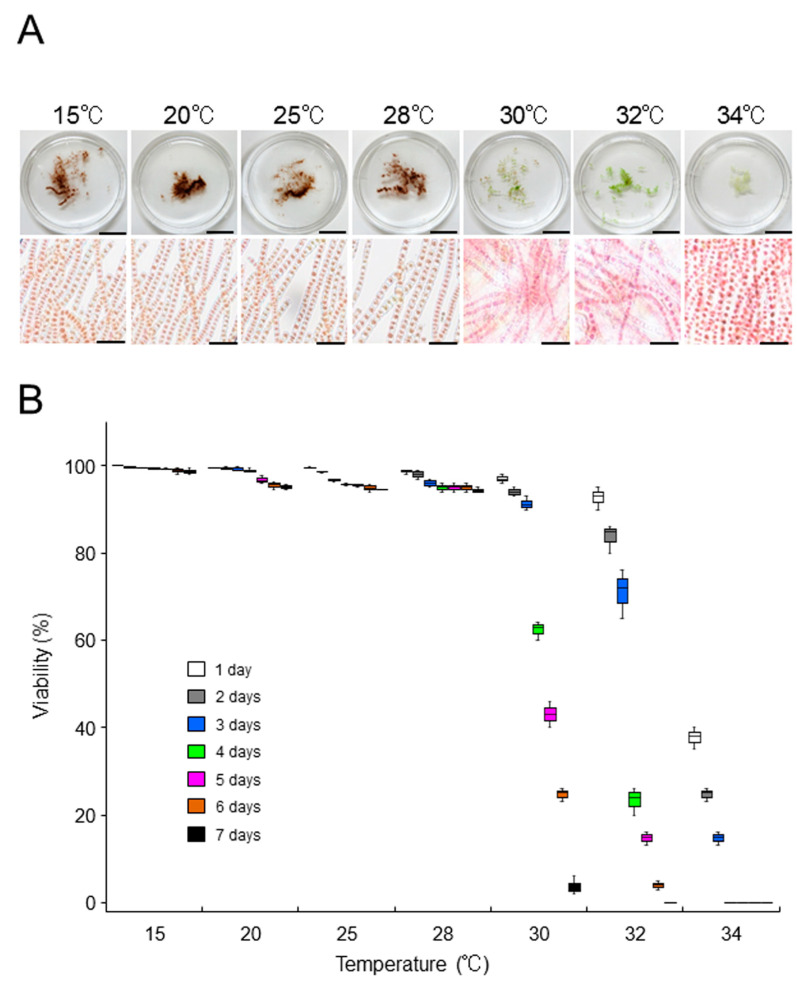
Effects of temperature on the viability of ‘*Bangia*’ sp. ESS2 thalli. Samples of the laboratory-maintained culture (0.05 g) were incubated at l5, 20, 25, 28, 30, 32, and 34 °C for 7 days, and changes in body color and viability of cells were observed: (**A**) Comparison of body color (upper panels) and staining pattern with erythrosine (lower panels) among thalli treated with various temperatures for 7 days. Scale bars, 1 cm in the upper panels and 50 μm in the lower panels; (**B**) Quantification of viability. Viability of thalli incubated at various temperatures was examined daily by staining with 0.01% erythrosine. Error bars indicate the standard deviation of triplicate experiments (*N* = 3).

When the thalli of ‘*Bangia*’ sp. ESS2 were incubated at 15, 20, 25, and 28 °C, they appeared dark red-brown. These thalli could not be stained with erythrosine (Figure 3A), and most vegetative cells were alive (Figure 3B and Table S1). In addition, their survival was not affected by a 3-day incubation at 30 °C or a 1-day incubation at 32 °C (Figure 2B). However, a 7-day incubation at 30, 32, or 34 °C promoted greening and staining of the thalli with erythrosine (Figure 3A), indicating the death of thalli. Indeed, viability gradually decreased depending on the duration of incubation, and over 95% of cells were dead after 7 days of culture (Figure 3B and Table S1). These results are different from our previous findings for ‘*Bangia*’ sp. ESS1 of the ‘*Bangia*’ 2 clade [12,23], which cannot survive at 32 °C, whereas 80 and 40% survival were observed following incubation at 30 °C for 7 days and 3 weeks, respectively [15].

**Figure 4:**
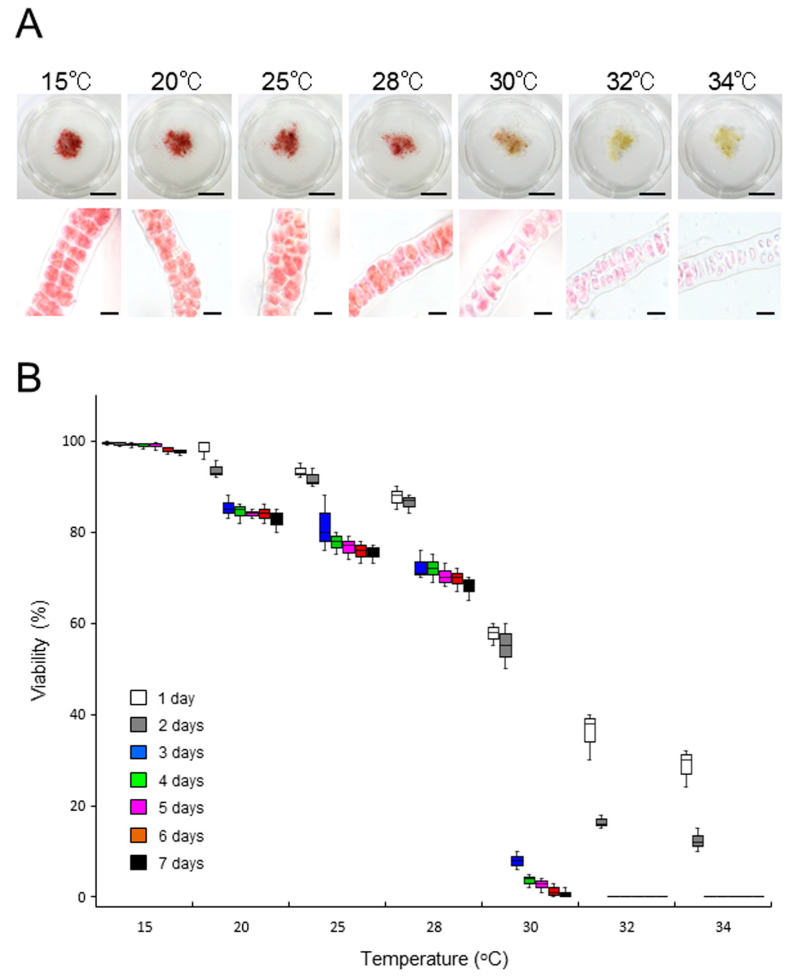
Effects of temperature on the viability of *Bangia atropurpurea* thalli. Samples of the laboratory-maintained culture (0.05 g) were incubated at 15, 20, 25, 28, 30, 32, and 34 °C for 7 days, and changes in body color and viability of cells were observed: (**A**) Comparison of body color (upper panels) and staining pattern with erythrosine (lower panels) among thalli treated with various temperature for 7 days. Scale bars, 1 cm in the upper panels and 25 μm in the lower panels; (**B**) Quantification of viability. Viability of thalli incubated at various temperatures was examined daily by staining with 0.01% erythrosine. Error bars indicate the standard deviation of triplicate experiments (*N* = 3).

By contrast, *B. atropurpurea* thalli were sensitive to temperatures >20 °C: 70–80% of thalli survived a 7-day incubation at 20, 25, and 28 °C but not at 30, 32, or 34 °C (Figure 4 and Table S3). These results indicate that *B. atropurpurea* is more sensitive to heat stress than ‘*Bangia*’ sp. ESS2 and that the level of intrinsic tolerance to heat stress in ‘*Bangia*’ sp. ESS1 is highest among the three species, although the growth-limiting temperatures of ‘*Bangia*’ sp. ESS1 and ‘*Bangia*’ sp. ESS2 are similar and slightly higher than that of *B. atropurpurea*. Therefore, sensitivity to heat stress varied among species from different ‘*Bangia*’ clades.

### 2.4. Repression of the Asexual Life Cycle by Heat Stress

**Figure 5:**
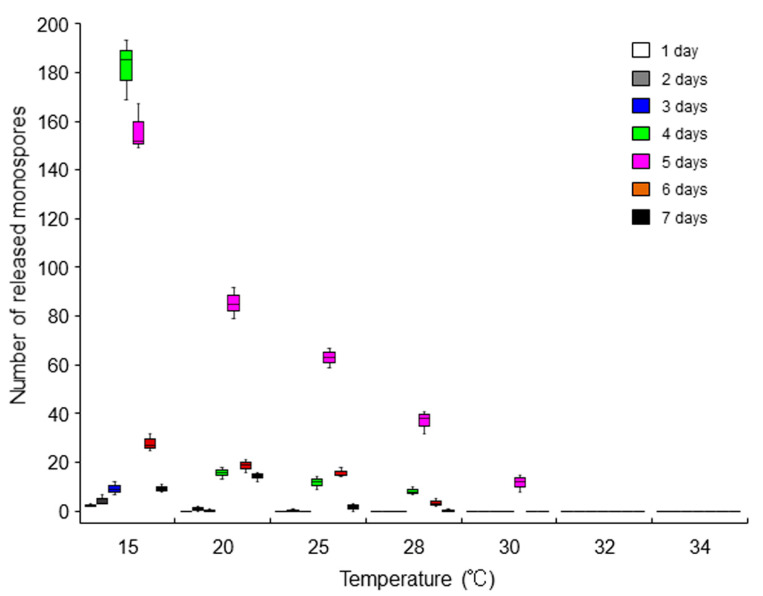
Effects of the heat stress on asexual propagation in ‘*Bangia*’ sp. ESS2. Release of asexual spores from thalli (0.02 g) cultured statically at l5, 20, 25, 28, 30, 32, and 34 °C was counted every day for 7 days. Error bars indicate the standard deviation of triplicate experiments (*N* = 3).

Since we previously observed the promotion of asexual sporulation at sublethal temperatures (such as 25 and 28 °C) in ‘*Bangia*’ sp. ESS1 [15], we examined the effects of heat stress on asexual spore release in ‘*Bangia*’ sp. ESS2 and *B. atropurpurea*. In ‘*Bangia*’ sp. ESS2, the maximum release of asexual spores was observed 4 and 5 days after starting static culture of thalli at 15 °C, whereas increasing the culture duration reduced the number of released spores (Figure 5 and Table S3). Unexpectedly, heat stress repressed the release of asexual spores. Asexual sporulation by 5-day cultures gradually decreased with increasing temperature, and no release was observed at 32 or 34 °C (Figure 5 and Table S3). Thus, the release of asexual spores in ‘*Bangia*’ sp. ESS2 is transient, with a peak at 4 and 5 days of static culture, and is repressed by heat treatment (Figure 5 and Table S3). By contrast, asexual reproduction was not observed in *B. atropurpurea* under heat-stress conditions.

The data in Figure 5 suggest that ‘*Bangia*’ sp. ESS2 responds to the loss of water movement (calm stress), which means a loss of hydrodynamic stress via the static culture in dishes without water moving, by accelerating spore release via a heat-repressive pathway, which is different from heat stress-enhanced spore release in ‘*Bangia*’ sp. ESS1 [15]. In fact, in ‘*Bangia*’ sp. ESS1, calm stress itself had only a small effect on promoting asexual sporulation [15]; however, exposure to calm stress and subsequent freezing stress highly stimulated spore release after thawing [24]. Therefore, the calm stress-dependent release of asexual spores is basically conserved between ‘*Bangia*’ sp. ESS1 and ‘*Bangia*’ sp. ESS2, although the roles of calm stress in regulating asexual spore release in these two species differ. In addition, ‘*Bangia*’ sp. ESS2 does not undergo heat stress-inducible sporulation (Figure 4 and Table S2), unlike ‘*Bangia*’ sp. ESS1 [15]. Based on these findings, we propose that the strategies for stress-dependent resetting of the timing of the asexual lifecycle are different between the ‘*Bangia*’ 2 and ‘*Bangia*’ 3 clades of Bangiales. Why the strategies for asexual spore release differ between ‘*Bangia*’ sp. ESS1 and ‘*Bangia*’ sp. ESS2, and how heat and calm stress differentially promote the asexual life cycle in a species-dependent manner remain to be elucidated.

### 2.5. Defect in the Acquisition of Heat-Stress Tolerance

**Figure 6:**
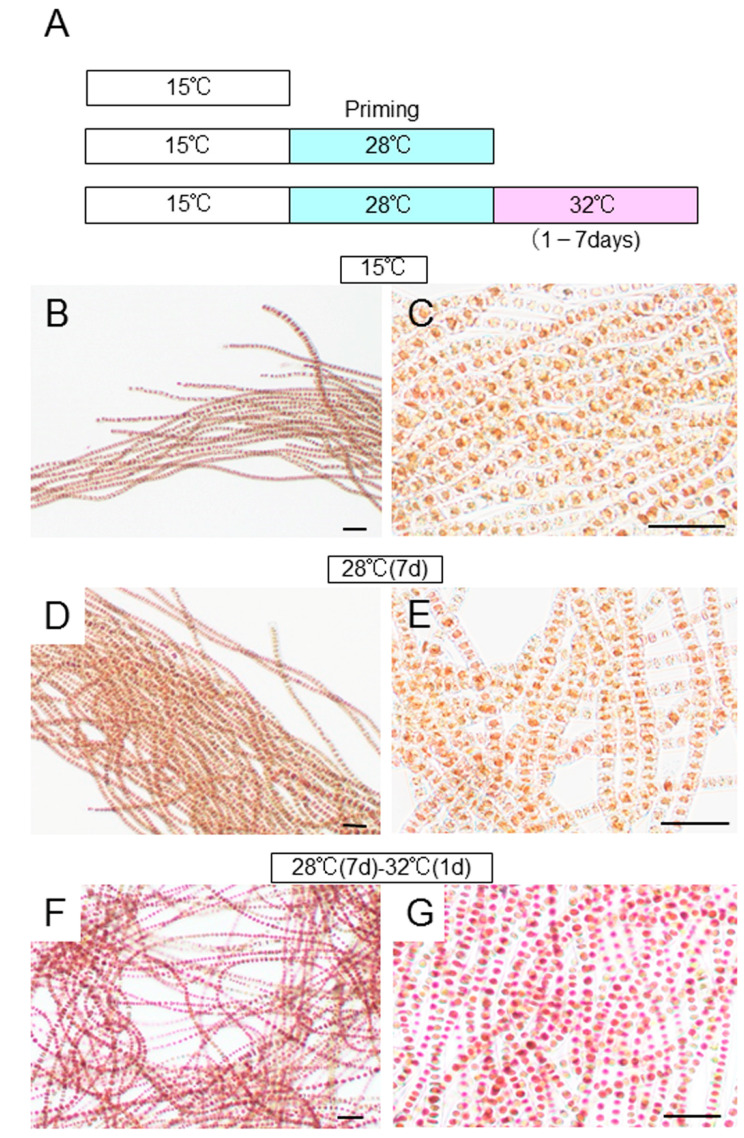
*‘Bangia’* sp. ESS2 thalli fail to acquire heat-stress tolerance: (**A**) Schematic representation of the experimental design to assess the ability of *‘Bangia’* sp. ESS2 to acquire heat-stress tolerance. Three temperature treatments were employed: control cultured at 15 °C, priming at 28 °C for 7 days, and treatment at 32 °C after priming for various durations from 1 to 7 days; (**B**,**C**) Photographs of control thalli grown at 15 °C shown at different magnifications; (**D**,**E**) Photographs of thalli primed at 28 °C for 7 days; (**F**,**G**) Photographs of thalli grown at 28 °C for 7 days, followed by 32 °C for 1 day. All thalli were stained with 0.01% erythrosine to visualize dead cells, which appear pink. Scale bars, 50 μm.

‘*Bangia*’ sp. ESS1 can acquire heat-stress tolerance by priming via incubation at non-lethal high temperatures, resulting in survival under subsequent exposure to lethal heat stress [16]. Thus, we addressed whether ‘*Bangia*’ sp. ESS2 and *B. atropurpurea* are also able to acquire heat-stress tolerance. We pre-incubated ‘*Bangia*’ sp. ESS2 thalli (Figure 6B,C) at 28 °C for 7 days, followed by incubation at 32 °C for 1 to 7 days (Figure 6A). Although the thalli were alive during pre-incubation at 28 °C (Figure 6D,E), as indicated in Figure 3, incubation at 32 °C for only 1 day killed all vegetative cells in the thalli (Figure 6F,G). These results demonstrate that ‘*Bangia*’ sp. ESS2 cannot acquire thermotolerance under sublethal heat-stress conditions.

**Figure 7:**
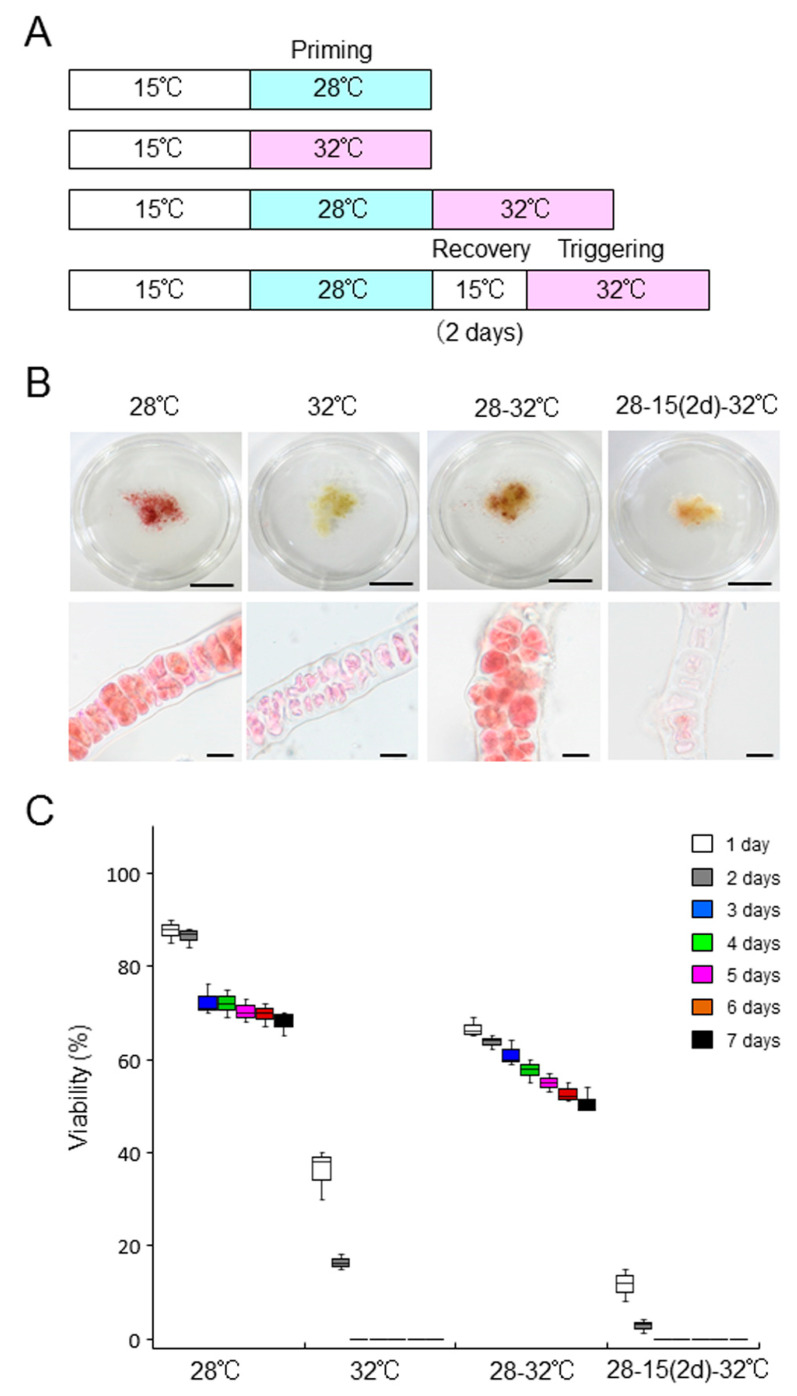
*Bangia atropurpurea* thalli fail to establish heat-stress memory: (**A**) Schematic representation of the experimental design to assess the ability of ‘*Bangia*’ sp. ESS2 to memorize heat stress. Four temperature treatments were employed: priming at 28 °C for 7 days, direct transfer to 32 °C from 15 °C, incubation at 32 °C for 7 days after priming at 28 °C, and treatment at 32 °C after recovery for 2 days; (**B**) Comparison of body color (upper panel) and staining pattern with erythrosine (lower panel) among thalli treated with various temperature conditions, as indicated. All thalli were stained with 0.01% erythrosine to visualize dead cells, which appear pink. Scale bars, 1 cm and 25 μm for upper and lower panels, respectively; (**C**) Quantification of viability. Viability of thalli incubated at various temperature conditions was visualized daily by staining with 0.01% erythrosine. Error bars indicate the standard deviation of triplicate experiments (*N* = 3).

We then tested the ability of *B. atropurpurea* to acquire thermotolerance. Although this alga showed approximately 70% survival at 28 °C, but not 32 °C after 7 days of culture (Figure 4 and Figure 7), the cultures were primed by treatment at 28 °C for 7 days, and the survival rate at the normally lethal temperature of 32 °C increased; for instance, a survival rate of 50% was observed after 7 days of culture at 32 °C (Figure 7, Table S4). Thus, *B. atropurpurea* can acquire thermotolerance by priming at 28 °C. We therefore examined whether *B. atropurpurea* can establish heat-stress memory to survive subsequent lethal high-temperature conditions. When the thalli were returned to 15 °C for 2 days after 7-day priming at 28 °C, incubation at 32 °C resulted in the loss of viability (Figure 7, Table S4). The results indicate that although *B. atropurpurea* has the ability to acquire thermotolerance by priming, it cannot establish heat-stress memory to maintain thermotolerance.

Since the living conditions of sessile organisms usually fluctuate dramatically and are often recurrent, the acquisition of thermotolerance via heat-stress memory (following exposure to sublethal heat-stress conditions) is thought to be essential for survival under subsequent lethal high-temperature stress in seaweeds including ‘*Bangia*’ sp. ESS1, as well as terrestrial plants [16,25,26,27,28]. Thus, the inability to acquire heat-stress tolerance in ‘*Bangia*’ sp. ESS2 and heat-stress memory in *B. atropurpurea* was unexpected; these are notable characteristics of poikilotherms. ‘*Bangia*’ sp. ESS1 and ‘*Bangia*’ sp. ESS2 were collected in May and April from different regions of the same island ([23] and see Section 3); thus, the environmental conditions experienced by these two species were likely similar. We propose that the different heat-stress response strategies of these species, rather than seasonal preferences, enable their compartmentalization on Kamomejima Island. In addition, *B. atropurpurea* was harvested from the rocky bed of a river (see Section 3); in such an environment, algae are usually splashed by river currents in the mountains and environmental conditions are relatively constant. Therefore, it is currently unclear whether the differences in the seasonal appearance of these species based on environmental conditions and their distinct heat-stress response strategies are related. Alternatively, since these three species belong to phylogenetically separated clades of ‘*Bangia*’, the variation in their heat-stress response strategies could be due to their different phylogenetic positions.

Despite these remaining unresolved questions, our results clearly indicate that species from different clades of ‘*Bangia*’ employ different heat-stress response strategies. Specifically, ‘*Bangia*’ sp. ESS2 and *B. atropurpurea* lack the intrinsic ability to acquire heat-stress tolerance and establish heat-stress memory to cope with recurrent changes in environmental conditions. Since this does not fit the general notion that poikilotherms require heat-stress tolerance and heat-stress memory [16,25,26,27,28], the presence of diverse responses to heat stress appears to be a special characteristic of ‘*Bangia*’ species. Therefore, it remains to be elucidated why ‘*Bangia*’ species from different clades employ different strategies in response to heat stress. Such information might help confirm the recently revised taxonomy of Bangiales [10].

## 3. Materials and Methods

### 3.1. Algal Materials, Culture Conditions, and Morphological Observation

Gametophytes of filamentous *Bangia* grown on rocks were harvested on April 20, 2018 from Kamomejima Island (41°52′ N, 140°06′ E) in Esashi, Hokkaido in Japan. The thalli were maintained in sterilized artificial seawater (SEALIFE, Marinetech, Tokyo, Japan) enriched with ESS2 [29] under 60–70 μmol photons m^−2^ s^−1^ light with a short-day photoperiod (10 h light/14 h dark) at 15 °C with air filtered through a 0.22 μm filter (Whatman, Maidstone, UK). Conchocelis filaments appeared during the culture of thalli and were maintained as described for thalli; the conchosporangia parasitically developed on the conchocelis filaments. The culture medium was changed weekly. Thalli of the freshwater species *Bangia atropurpurea*, which were collected from the rocky bed of a river with a rapid current in Higashi-kawachisawa, Shizuoka, Japan in May 2005 and April 2006, were maintained according to Yokono et al. [17] in commercially available Ca^2+^-rich mineral water (Contrex^®^, Nestlé Waters Marketing & Distribution) in plastic culture vessels, except that the culture conditions described above for marine *Bangia* species were utilized. Thalli, conchocelis, and conchosporangia were observed and imaged under an Olympus IX73 light microscope (Olympus, Tokyo, Japan) equipped with an Olympus DP22 camera.

### 3.2. Phylogenetic Analysis

Total genomic DNA was extracted from the species collected at Esashi from air-dried samples using a DNeasy Plant Mini kit (Qiagen, Valencia, CA, USA) according to the manufacturer’s instructions. A 466-bp portion of the *rbcL* gene was amplified from the Esashi species with gene-specific primers (5′-AAGTGAACGTTACGAATCTGG-3′ and 5′-GATGCTTTATTTACACCCT-3′; [30]) using Ex Taq polymerase (TaKaRa Bio, Kusatsu, Japan) and sequenced on an ABI Model 3130 Genetic Analyzer (Life Technologies, Carlsbad, CA, USA). The nucleotide sequence of the amplified DNA fragment was deposited in DDBJ/EMBL/GenBank under accession number LC602264. A neighbor-joining phylogenetic tree was constructed with MEGA 7 software (https://www.megasoftware.net (accessed on 2 April 2021) using ClustalW to align the *rbcL* sequences from other *Bangia* species [10]. The accession numbers of these *rbcL* sequences are listed in front of the species’ names in the phylogenetic tree.

### 3.3. Determining the Growth-Limiting Temperature

Each 0.05 g sample (fresh weight) of thalli (from aeration cultures grown at 15 °C) was incubated statically in dishes (Azunoru dish; 90 mm diameter × 20 mm height, As One Co., Ltd.) containing 50 mL of seawater for marine species or Contrex^®^ for freshwater species at 20, 25, 28, 30, 32, and 34 °C for 7 days, while control experiments were performed at 15 °C. The viability of these thalli was visualized daily by staining with artificial seawater containing 0.01% erythrosine (Wako Pure Chemical Industries, Japan) as described by Kishimoto et al. [16]. In brief, thalli were stained for 5 min at room temperature, gently rinsed with artificial seawater or Contrex^®^ to remove excess erythrosine, and mounted on a slide with medium. The thalli were observed and photographed under an Olympus IX73 light microscope equipped with an Olympus DP22 camera. Cells stained by the dye were defined as dead cells. Viability was calculated from the number of living and dead cells obtained using micrographs. Analysis of samples under each treatment condition was repeated three times.

### 3.4. Quantification of Released Asexual Spores

Each 0.02 g sample (fresh weight) of thalli (aeration-cultured at 15 °C) was incubated statically in dishes (Azunoru dish; 90 mm diameter × 20 mm height, As One) containing 50 mL of seawater at 15, 20, 25, 28, 30, 32, and 34 °C for 7 days. The number of asexual spores released onto the bottom of the dishes was counted daily by observation under an Olympus IX73 light microscope. Analysis of samples under each treatment condition was repeated three times.

### 3.5. Confirmation of Acquisition of Thermotolerance

Each 0.05 g (fresh weight) of thalli aeration-cultured at 15 °C was incubated statically in dishes (Azunoru dish; 90 mm diameter × 20 mm height, As One) containing 50 mL of seawater at 28 °C for 7 days or 28 °C for 7 days plus subsequent treatment at 32 °C for 1 to 7 days. The viability of these thalli was examined as described above. Analysis of samples under each treatment condition was repeated three times.

### 3.6. Statistical Analysis

Values are indicated by ±SD from triplicate experiments. Two-way ANOVA followed by a Tukey–Kramer test was used for multiple comparisons, and significant differences were determined using a cutoff value of *p* < 0.05.

## 4. Conclusions

We identified ‘*Bangia*’ sp. ESS2 as a member of ‘*Bangia*’ 3 and compared its heat-stress response strategies with those of *B. atropurpurea* and ‘*Bangia*’ sp. ESS1. Our analysis revealed diversity in the heat-stress response strategies among these three species in terms of asexual sporulation, the acquisition of thermotolerance, and the memorization of heat stress. These findings suggest that the intrinsic abilities to respond to and tolerate heat stress vary among species from different clades of ‘*Bangia*’. Physiological and molecular biological studies of the mechanisms regulating heat-stress responses and the memorization of heat stress in species from inter- and intra-‘*Bangia*’ clades could help explain why these strategies are diverse in ‘*Bangia*’. Such information would increase our understanding of the biology of ‘*Bangia*’ species from different clades and could confirm the recently revised taxonomy of Bangiales.

## Data Availability

Data are contained within the article.

## Supplementary Materials

The following are available online at https://www.mdpi.com/2223-7747/10/8/1733/s1, Table S1: Viability of vegetative cells in ‘*Bangia*’ sp. ESS2 thalli in response to different durations of incubation under various temperature conditions, Table S2: Viability of vegetative cells in *Bangia atropurpurea* thalli in response to different durations of incubation under various temperature conditions, Table S3: Number of asexual spores released from ‘*Bangia*’ sp. ESS2 thalli in response to different durations of incubation under various temperature conditions, Table S4: Viability of vegetative cells in *Bangia atropurpurea* thalli associated with the acquisition of heat-stress tolerance and the memorization of heat stress.
