# Supplementary material for: Heat-Stress Responses Differ among Species from Different ‘Bangia’ Clades of Bangiales (Rhodophyta)
Source: Plants (Basel). 2021 Aug 22;10(8):1733. doi: 10.3390/plants10081733 (PMC8412102; doi:10.3390/plants10081733)
Supplement: Supplementary file 1 [file plants-10-01733-s001.zip › Table S1 rev.pdf]

Table S1. Viability of vegetative cells in ‘*Bangia*’ sp. ESS2 thalli in response to different durations of incubation under various temperature conditions

Duration (Days)	Temperature						
	15°C	20°C	25°C	28°C	30°C	32°C	34°C
1	100 ± 0 [a]	99.5 ± 0.1 [abc]	99.57 ± 0.21 [abc]	98.67 ± 0.58 [abcd]	97 ± 1 [bcdef]	92.67 ± 2.52 [hi]	37.67 ± 2.52 [n]
2	99.67 ± 0.21 [ab]	99.37 ± 0.38 [abc]	98.53 ± 0.12 [abcd]	98 ± 1 [abcde]	94 ± 1 [gh]	83.67 ± 3.21 [j]	24.67 ± 1.53 [o]
3	99.53 ± 0.12 [abc]	99.27 ± 0.46 [abc]	96.67 ± 0.23 [cdefg]	96 ± 1 [defg]	91.33 ± 1.53 [i]	71 ± 5.57 [k]	14.67 ± 1.53 [p]
4	99.4 ± 0.2 [abc]	98.9 ± 0.44 [abc]	95.7 ± 0.26 [efg]	95 ± 1 [fgh]	62.33 ± 2.08 [l]	23.33 ± 3.06 [o]	0 ± 0 [r]
5	99.3 ± 0.17 [abc]	96.8 ± 0.98 [bcdef]	95.5 ± 0.36 [efg]	95 ± 1 [fgh]	43 ± 3 [m]	14.67 ± 1.53 [p]	0 ± 0 [r]
6	98.87 ± 0.81 [abc]	95.53 ± 0.83 [efg]	94.87 ± 0.9 [fgh]	95 ± 1 [fgh]	24.67 ± 1.53 [o]	4 ± 1 [q]	0 ± 0 [r]
7	98.7 ± 0.82 [abc]	95.17 ± 0.51 [fgh]	94.47 ± 0.12 [fgh]	94.33 ± 0.58 [fgh]	3.67 ± 2.08 [q]	0 ± 0 [r]	0 ± 0 [r]

Mean values ± SD (%) per 0.05 g sample fresh weight were calculated from triplicate experiments and letters denote statistically significant differences ($p < 0.05$) as determined by the Tukey-Kramer test following two-way ANOVA.
