# Supplementary material for: Heat-Stress Responses Differ among Species from Different ‘Bangia’ Clades of Bangiales (Rhodophyta)
Source: Plants (Basel). 2021 Aug 22;10(8):1733. doi: 10.3390/plants10081733 (PMC8412102; doi:10.3390/plants10081733)
Supplement: Supplementary file 1 [file plants-10-01733-s001.zip › Table S2 rev.pdf]

Table S2. Viability of vegetative cells in *Bangia atropurpurea* thalli in response to different durations of incubation under various temperature conditions

Duration (Days)	Temperature (°C)						
	15°C	20°C	25°C	28°C	30°C	32°C	34ºC
1	99.5 ± 0.44 ^{a}	98.4 ± 2.08 ^{ab}	93.33 ± 1.53 ^{abc}	87.67 ± 2.52 ^{cde}	57.67 ± 2.52 ^{i}	36 ± 5.29 ^{m}	28.67 ± 4.16 ^{m}
2	99.33 ± 0.38 ^{a}	93.57 ± 1.91 ^{abc}	91.67 ± 2.08 ^{bcd}	86.33 ± 2.08 ^{cde}	55 ± 5 ^{i}	16.33 ± 1.53 ^{n}	12.33 ± 2.52 ^{n}
3	99.17 ± 0.49 ^{ab}	85.33 ± 2.52 ^{de}	81.33 ± 6.11 ^{efgh}	72.33 ± 3.21 ^{ijk}	8 ± 2 ^{op}	0 ± 0 ^{q}	0 ± 0 ^{q}
4	99 ± 0.61 ^{ab}	84.33 ± 2.08 ^{def}	77.67 ± 2.52 ^{fghi}	72 ± 3 ^{ijk}	3.67 ± 1.53 ^{pq}	0 ± 0 ^{q}	0 ± 0 ^{q}
5	98.97 ± 0.84 ^{ab}	84 ± 1 ^{efg}	76.67 ± 2.52 ^{ghij}	70.33 ± 2.52 ^{ijk}	2.67 ± 1.53 ^{pq}	0 ± 0 ^{q}	0 ± 0 ^{q}
6	98.03 ± 0.9 ^{ab}	84 ± 2 ^{efg}	75.67 ± 2.52 ^{hij}	69.67 ± 2.52 ^{ijk}	1.33 ± 1.53 ^{pq}	0 ± 0 ^{q}	0 ± 0 ^{q}
7	97.5 ± 0.61 ^{ab}	82.67 ± 2.52 ^{efgh}	75.33 ± 2.08 ^{hijk}	68 ± 2.65 ^{jk}	0.67 ± 1.15 ^{pq}	0 ± 0 ^{q}	0 ± 0 ^{q}

Mean values ± SD (%) per 0.05 g sample fresh weight were calculated from triplicate experiments and letters denote statistically significant differences ($p < 0.05$) as determined by the Tukey-Kramer test following two-way ANOVA.
