# Supplementary material for: Heat-Stress Responses Differ among Species from Different ‘Bangia’ Clades of Bangiales (Rhodophyta)
Source: Plants (Basel). 2021 Aug 22;10(8):1733. doi: 10.3390/plants10081733 (PMC8412102; doi:10.3390/plants10081733)
Supplement: Supplementary file 1 [file plants-10-01733-s001.zip › Table S4 rev.pdf]

Table S4. Viability of vegetative cells in *Bangia atropurpurea* thalli associated with the acquisition of heat stress tolerance and the memorization of heat stress

Duration	Experiment conditions			
(Days)	28°C	32°C	28-32°C	28-15(2d)-32°C
1	87.67 ± 2.52 [a]	36 ± 5.29 [i]	66.67 ± 2.08 [cd]	11.67 ± 3.51 [j]
2	86.33 ± 2.08 [a]	16.33 ± 1.53 [j]	63.67 ± 1.53 [cde]	2.67 ± 1.53 [k]
3	72.33 ± 3.21 [b]	0 ± 0 [k]	61 ± 2.65 [def]	0 ± 0 [k]
4	72 ± 3 [b]	0 ± 0 [k]	57.67 ± 2.52 [efg]	0 ± 0 [k]
5	70.33 ± 2.52 [bc]	0 ± 0 [k]	55 ± 2 [fgh]	0 ± 0 [k]
6	69.67 ± 2.52 [bc]	0 ± 0 [k]	52.67 ± 2.08 [gh]	0 ± 0 [k]
7	68 ± 2.65 [bc]	0 ± 0 [k]	50.67 ± 2.89 [h]	0 ± 0 [k]

Incubation at various temperature was performed for 7 days, except for recovering at 15°C for 2 days. Mean values ± SD (%) per 0.05 g sample fresh weight were calculated from triplicate experiments and letters denote statistically significant differences ($p < 0.05$) as determined by the Tukey-Kramer test following two-way ANOVA.
