# Supplementary material for: Heat-Stress Responses Differ among Species from Different ‘Bangia’ Clades of Bangiales (Rhodophyta)
Source: Plants (Basel). 2021 Aug 22;10(8):1733. doi: 10.3390/plants10081733 (PMC8412102; doi:10.3390/plants10081733)
Supplement: Supplementary file 1 [file Table S3 rev.pdf › Table S3 rev.pdf]

Table S3. Number of asexual spores released from '*Bangia*' sp. ESS2 thalli in response to different durations of incubation under various temperature conditions

Duration	Temperature						
(Days)	15°C	20°C	25°C	28°C	30°C	32°C	34°C
1	2.33 ± 0.58^{ab}	0 ± 0^{a}	0 ± 0^{a}	0 ± 0^{a}	0 ± 0^{a}	0 ± 0^{a}	0 ± 0^{a}
2	4.33 ± 2.31^{ab}	1 ± 1^{a}	0.33 ± 0.58^{a}	0 ± 0^{a}	0 ± 0^{a}	0 ± 0^{a}	0 ± 0^{a}
3	9.33 ± 2.52^{abcd}	0.33 ± 0.58^{a}	0 ± 0^{a}	0 ± 0^{a}	0 ± 0^{a}	0 ± 0^{a}	0 ± 0^{a}
4	182.33 ± 12.22^{j}	15.67 ± 2.52^{cd}	11.67 ± 2.52^{bcd}	8.33 ± 1.53^{abc}	0 ± 0^{a}	0 ± 0^{a}	0 ± 0^{a}
5	156 ± 9.64^{i}	85.33 ± 6.51^{h}	63 ± 4^{g}	37 ± 4.58^{f}	11.67 ± 3.51^{bcd}	0 ± 0^{a}	0 ± 0^{a}
6	28 ± 3.6^{ef}	18.67 ± 2.52^{e}	15.67 ± 2.08^{cd}	3.33 ± 1.53^{ab}	0 ± 0^{a}	0 ± 0^{a}	0 ± 0^{a}
7	9.33 ± 1.53^{abcd}	14.33 ± 2.08^{cd}	1.67 ± 1.53^{a}	0.33 ± 0.58^{a}	0 ± 0^{a}	0 ± 0^{a}	0 ± 0^{a}

Mean values ± SD (number of asexual spore release) per 0.02 g sample fresh weight were calculated from triplicate experiments and letters denote statistically significant differences ($p < 0.05$) as determined by the Tukey-Kramer test following two-way ANOVA.
